# Cancer-Associated Fibroblasts Exposed to High-Dose Ionizing Radiation Promote M2 Polarization of Macrophages, Which Induce Radiosensitivity in Cervical Cancer

**DOI:** 10.3390/cancers15051620

**Published:** 2023-03-06

**Authors:** Yuhan Sheng, Baofang Zhang, Biyuan Xing, Zhao Liu, Yu Chang, Gang Wu, Yingchao Zhao

**Affiliations:** Cancer Center, Union Hospital, Tongji Medical College, Huazhong University of Science and Technology, Wuhan 420022, China

**Keywords:** cervical cancer, radiotherapy, radioresistance, tumor-associated macrophages, cancer-associated fibroblasts

## Abstract

**Simple Summary:**

Tumor-associated macrophages (TAMs) and cancer-associated fibroblasts (CAFs) in the tumor microenvironment are critical factors in the curative effects of cancer therapies. The aim of our study was to explore the interactions between TAMs and CAFs in the context of ionizing radiation. We confirmed that M2 macrophages correlate with poor prognosis and induce radioresistance in cervical cancer. M2 polarization was increased after high-dose IR in both mouse models and patients with cervical cancer. M2 macrophage contents were increased in CAF-positive regions in patients with cervical cancer who relapsed after receiving radical radiotherapy. In addition, high-dose irradiated CAFs promote macrophage M2 polarization in cervical cancer through the secretion of chemokine (C-C motif) ligand 2 (CCL2). Our data provide a new insight into the relation between CAFs and TAMs under IR, which is of significance for further exploration of the mechanism of radioresistance in cervical cancer.

**Abstract:**

Radiotherapy, including brachytherapy, is a major therapeutic regimen for cervical cancer. Radioresistance is a decisive factor in radiation treatment failure. Tumor-associated macrophages (TAMs) and cancer-associated fibroblasts (CAFs) in the tumor microenvironment are critical factors in the curative effects of cancer therapies. However, the interactions between TAMs and CAFs in the context of ionizing radiation are not fully understood. This study was undertaken to investigate whether M2 macrophages induce radioresistance in cervical cancer and to explore the TAMs’ phenotypic transformation after IR and its underlying mechanisms. The radioresistance of cervical cancer cells was enhanced after being co-cultured with M2 macrophages. TAMs tended to undergo M2 polarization after high-dose irradiation, which was strongly associated with CAFs in both mouse models and patients with cervical cancer. Additionally, cytokine and chemokine analysis was performed to find that high-dose irradiated CAFs promoted macrophage polarization towards the M2 phenotype through chemokine (C-C motif) ligand 2. Collectively, our results highlight the crucial role that high-dose irradiated CAFs play in the regulation of M2 phenotype polarization, which ultimately induces radioresistance in cervical cancer.

## 1. Introduction

Cervical cancer is the most common gynecologic cancer and the main cause of cancer-related mortality among women in developing countries [1]. Worldwide, more than 5,000,000 women are diagnosed with cervical cancer and 300,000 die because of the disease each year [2]. Brachytherapy, in which radioactive materials are placed in or near the tumor and high-dose ionizing radiation (IR) is delivered directly to the lesion, is a routine cervical cancer treatment [3]. Therefore, both external-beam radiation therapy (EBRT) and brachytherapy with low segmentation and a high dose per fraction could lead to the tolerance to radiotherapy of cervical cancer. However, mainly because of local recurrences, three-year progression-free survival (PFS) after radiotherapy remains unsatisfactory [4,5,6]. Therefore, exploring effective methods to improve the radiosensitivity of cervical cancer is urgently needed.

IR triggers treatment responses in components within the tumor microenvironment (TME) other than cancer cells [7]. On the one hand, the secretion of inflammatory cytokines and exposed antigens after IR promote the maturation and activation of dendritic cells (DCs), which mediate T-cell responses against cancer cells. On the other hand, IR stimulates immunosuppressive cells, such as the regulatory T cells and myeloid-derived suppressor cells [8]. Therefore, exploring the immunoregulatory effect of IR may help to shed light on radiosensitization [7]. Macrophages are among the most abundant cells in the TME and are classified into two categories: M1-type macrophages, which exert antitumor effects, and M2-type macrophages, which have pro-tumoral activities. The content of M2 macrophages has been found to be correlated with poor prognosis in various cancers, including cervical cancer [9,10]. Several studies have demonstrated that high-dose radiation induces the phenotypic transformation of tumor-associated macrophages (TAMs); however, the results are inconsistent [11,12,13]. Therefore, macrophage phenotype transformation after IR and its relationship with radiosensitivity in cervical cancer remains unknown. 

The mechanisms underlying radioresistance in cervical cancer may differ from those in other cancers because of the specific type of radiation used, i.e., internal radiation, which is usually performed in the later stages of treatment when the tumor burden is low. This indicates that the protection delivered from stroma to tumor tissue may be the reason for the tolerance of internal radiation therapy. Fibroblasts are a major component of the tumor stroma which is known to promote tumor growth and progression, and recent studies have shown a correlation between the distribution of fibroblasts and macrophages in various cancers [14,15], and that fibroblasts can induce M2 macrophage polarization through the secretion of interleukin 6 and stromal cell-derived factor 1 [16]. To our acknowledge, the effect of cancer-associated fibroblasts (CAFs) on TAMs under IR in cervical cancer has not been reported. 

Our objective was to assess the effect of M2 macrophages on prognosis and radioresistance in cervical cancer and to assess the TAMs’ phenotypic transformation after IR and its underlying mechanisms.

## 2. Materials and Methods

### 2.1. Specimens

In total, 151 tumor biopsy specimens were obtained from patients with cervical cancer at the Cancer Center, Union Hospital, Tongji Medical College, Huazhong University of Science and Technology, between March 2014 and June 2018. All patients received radical external radiation therapy at a dose of 45–50.4 Gy/25–28 F followed by brachytherapy at a dose of 21–35 Gy/3–5 F alone or combined with chemotherapy, and outcomes were followed up. Samples were collected from all patients before they received radiation therapy, and five samples were obtained from relapse lesions after radiotherapy. All patients provided informed consent. The study was approved by the institutional review board of Wuhan Union Hospital.

### 2.2. Immunohistochemical Staining

Tumor tissues were fixed with 4% paraformaldehyde immediately after resection and embedded in paraffin. Immunohistochemical staining was performed according to standard procedures, and the locations of CAFs and TAMs were evaluated in serial sec-tions. The primary antibodies used were anti-α-SMA (ab5694; Abcam, Cambridge, UK), anti-CD163 (ab182422; Abcam), and anti-CD206 (ab64693; Abcam). For M2 macrophage quantification, the samples were viewed at high magnification (400×) in five randomly selected regions. Macrophages were counted using ImageJ software, and averages were calculated. For the quantification of M2 macrophages around CAFs, the samples were viewed at low magnification (100×), and the region with the largest area of positively stained CAFs (hotspot area) was selected. M2 macrophages in this region were quantified as described above. 

### 2.3. Cell Culture

The human cell lines HeLa, SiHa, CaSki, and THP-1 were purchased from the Cell Bank of the Chinese Academy of Sciences (Shanghai, China) and were cultured in Dul-becco’s modified Eagle’s medium (DMEM) or RPMI-1640 culture medium supplemented with 10% fetal bovine serum. 

To generate macrophages, cells were isolated from the bone marrow of wild-type C57BL/6 mice or human peripheral blood (mononuclear cells were isolated from buffy coats by density gradient using Ficoll and suspended for 1 h to allow monocyte adherence and remove non-adherent cells. The cells were cultured in the presence of 20 ng/mL macrophage colony stimulating factor (M-CSF; PeproTech, Rocky Hill, NJ, USA) for 7 days to generate M0 macrophages. For THP-1 derived macrophages, 5 * 105/mL THP-1 cells were treated with 200 ng/mL phorbol 12-myristate 13-acetate (PeproTech) for 24 h to become M0 macro-phages. M0 macrophages were treated with 100 ng/mL lipopolysaccharide (Sigma, St. Louis, MO, USA) + 20 ng interferon-γ (PeproTech) or 20 ng/mL IL-4 (PeproTech) + 20 ng/mL IL-13 (PeproTech) for 24 h to generate M1 and M2 macrophages, respectively.

The reagents used for the experiments included rm-CCL2, rh-CCL2 (PeproTech), pe-roxisome proliferator-activated receptor (PPAR)γ antagonist (GW9662; MCE), CCL2 re-ceptor (CCR2) antagonist (INCB3344; MCE), and CCL2-neutralizing antibody and isotype control IgG (Clone2H5; eBioscience, Vienna, Austria). 

### 2.4. CAF Extraction

Tumor tissues were washed with sterile phosphate-buffered saline to remove necrotic tissue and blood. Then, they were cut up with scissors as much as possible, completely minced, and digested in culture medium supplemented with 0.1% collagenase at 37 °C. The suspension was passed through a 0.4-μm filter to obtain a single-cell suspension. The cells were incubated at 37 °C for 30 min to allow CAF adherence. The supernatant containing non-attached cells was discarded, and the remaining cells were used as pure CAFs. Cells of less than 10 passages were used in the experiments.

### 2.5. Construction of a Co-Culture SYSTEM

A co-culture system was established using 0.4-μm Transwell inserts (Corning, NY, USA) in a 6-well culture plate. For the co-culture of macrophages and cancer cells, macrophages were seeded on the upper layer and cancer cells were seeded on the bottom layer (at 60–70% confluence), and the cells were cultured for 24 h. For the co-culture of macrophages and CAFs, CAFs were seeded on the upper layer (at 30–40% confluence) and macrophages were seeded on the bottom layer, and the cells were cultured for 3 days. 

### 2.6. Colony Formation Assay

Cervical cancer cells were seeded in 6-well plates at different concentrations according to the indicated IR dose and were cultured in fresh culture medium for 10–14 days. Clones were fixed with 4% paraformaldehyde for 30 min and stained with a 0.1% crystal violet solution. The adherence rate was calculated as the number of clones/number of cells seeded × 100%. The survival fraction was calculated as the adhesion rate at dose Y in group X/adhesion rate at 0 Gy in group X.

### 2.7. Apoptosis Assay

Apoptosis was assayed using the Annexin V-FITC Apoptosis Detection Kit (C1062; Beyotime, Shanghai, China) following the manufacturer’s instructions. The cells were assessed by flow cytometry within 1 h.

### 2.8. Cell Cycle Arrest Assay

Cells were collected and fixed in 75% alcohol at −20 °C overnight. The cells were incubated with propidium iodide and RNase (Beyotime) in the dark for 30 min and as-sessed by flow cytometry.

### 2.9. Flow Cytometry 

Cells were stained with LIVE/DEAD Cell Viability Assay kit agents (65-0868-14; eBioscience) and blocked with 5 μg/mL anti-mouse CD16/32 (14-0161-81; eBioscience) before surface staining. Cells were stained for surface markers F4/80 (clone BM8) (11-4801-81; eBioscience), Gr-1 (108427; BioLegend, San Diego, CA, USA), CD45 (25-0451-81; eBioscience), CD11b (101263; BioLegend), and CD86 (105007; BioLegend) on ice. Then, the cells were fixed with 2% paraformaldehyde (Sigma-Aldrich), permeabilized with permeabilization buffer (BioLegend), and stained with directly labeled antibody to CD206 (MMR) (141707; BioLegend). 

### 2.10. Phagocytosis Assay

Lewis cancer cells were incubated with 5 nM carboxyfluorescein succinimidyl ester (CFSE) at 37 °C in the dark for 20 min. After washing away free CFSE, the CFSE-labeled Lewis cells were collected, and the cell concentration was adjusted. The collected cells were added to bone marrow-derived macrophages (BMDMs) at a tumor cell:macrophage ratio of 1:4 and cultured for 2 h. The cells were then incubated with F4/80-FITC (clone BM8, BioLegend) for flow cytometry analysis. 

### 2.11. Cytokine Profiling

The supernatant of CAFs and cervical cancer cells was assayed for cytokines and chemokines using the Mouse XL Cytokine Array (Ary028; R&D Systems) and CCL2 ELISA kits (mouse, EK0568; human, EK0441; BOSTER) according to the manufacturers’ instructions.

### 2.12. Quantitative Reverse Transcription (RT-q)PCR Analysis

Total RNA was isolated using TRIzol (TaKaRa, Tokyo, Japan) and reverse transcribed into cDNA using PrimeScript™ RT Master Mix (TaKaRa). qPCRs were run in a 20-μL reaction mixture using TB Green™ Premix Ex Taq™ II (Tli RNaseH Plus) (TaKaRa). GAPDH was used as an endogenous control. The primers used are listed in Table S1.

### 2.13. Western Blotting 

Cells were lysed using RIPA NP-40 RIPA Lysis buffer containing phenylmethylsulfonyl fluoride and protease inhibitor. The proteins (20–50 μg) were separated by 8–10% precast sodium dodecyl-sulfate polyacrylamide gel electrophoresis at 80 V and then blotted onto polyvinylidene difluoride membranes at 200 mA for a suitable time. The membranes were blocked and probed with primary antibodies, including anti-GAPDH (5174; Cell Signaling Technology (CST)), anti-CD163 (ab182422; Abcam), anti CD206/MRC-1 (ab64693; Abcam), anti-Akt (4685; CST), anti-pAkt (Ser473) (4060; CST), anti-Erk1/2 (4695; CST), anti-pErk1/2 (4370; CST), and anti-PPARγ (ab45036; Abcam) antibodies. After incubation with HRP-conjugated antibodies specific for rabbit or mouse IgG, the blots were developed using ECL chemiluminescence reagent. Bands were quantified using ImageJ. 

### 2.14. Immunofluorescence Analysis

Cells cultured on slides were fixed with 4% paraformaldehyde. After permeabilization with 0.25% Triton X-100 for 10 min, the cells were incubated with primary anti-bodies (anti γ-H2AX (2718; CST) and anti Ym-1 (192029; Abcam) at 4 °C overnight. Then, the cells were incubated with secondary antibodies diluted in blocking buffer in the dark at room temperature. Finally, the cells were stained with DAPI and observed using a fluorescence microscope.

### 2.15. Tumor Xenograft Mouse Models

Siha (5 × 105) or HeLa (5 × 105) cervical cancer cells were implanted subcutaneously into 6–8-week-old female BALB/c-Nu mice. Tumor volumes were measured every 3 days (tumor volume = length × width^2^ × 0.5). When tumor volumes reached approximately 100 mm^3^, the mice were randomly assigned to indicated groups (n = 8 per group) before treatment initiation. The study was approved by the Animal Management Committee of Huazhong University of Science and Technology.

### 2.16. Radiation Parameters

Cells seeded in culture dishes were irradiated with a single dose of 2, 4, 6, and 8 Gy. Mice in IR groups were anesthetized with sodium pentobarbital (60 mg/kg intraperitoneally), and the right posterior limbs (with tumors) were subjected to local IR using an X-ray irradiator (Varian, Palo Alto, CA, USA) at a beam energy of 6 MV and dose rate of 6 Gy/min.

### 2.17. Statistical Analysis

All experimental data were processed using SPSS 23.0 and GraphPad Prism 7 software and are presented as mean ± standard deviation (SD). The data were statistically analyzed using Student’s *t*-test, one-way ANOVA or chi-square test as appropriate. Kaplan–Meier analysis was conducted, using the log-rank test to determine statistical significance. A value of *p* < 0.05 was considered statistically significant.

## 3. Results

### 3.1. M2 Macrophages Correlate with Radiosensitivity 

**Figure 1:**
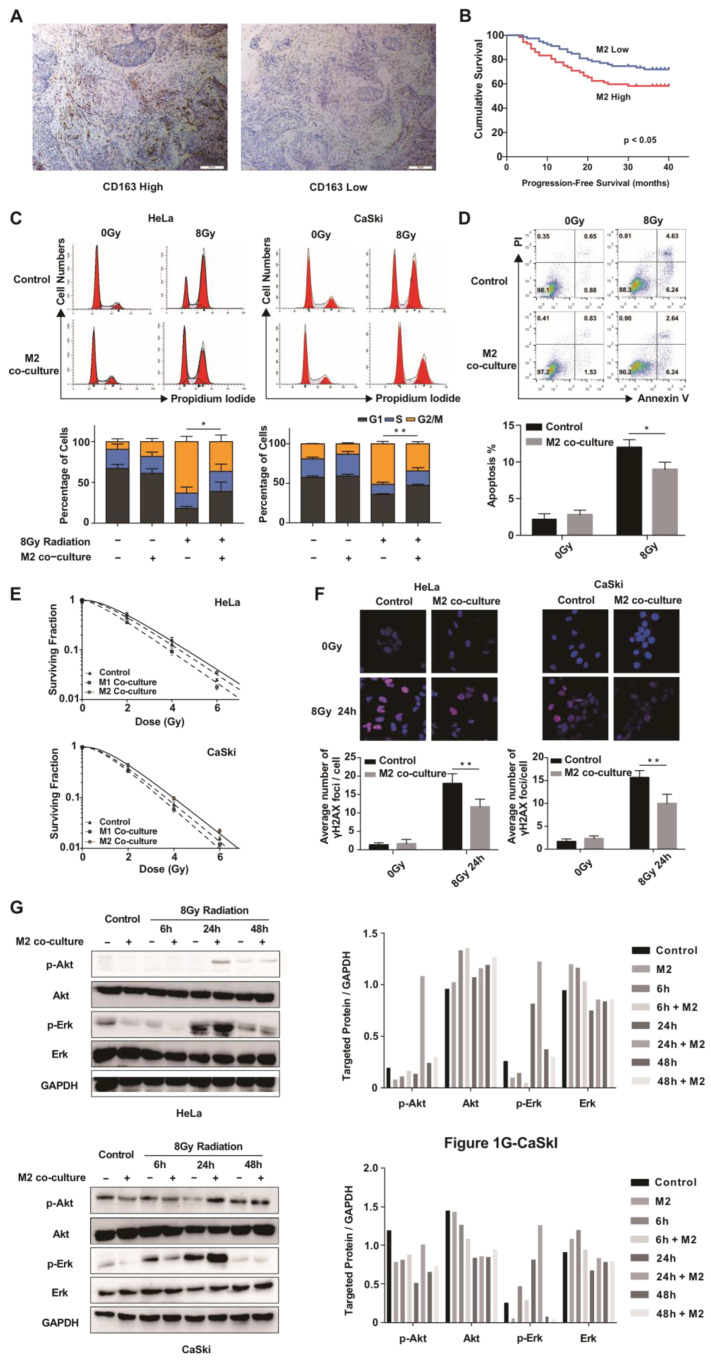
M2 macrophages correlate with radiosensitivity in cervical cancer. (**A**) Representative staining of CD163^+^ high expression (**left**) and CD163^+^ low expression (right) in patients with cervical cancer. (**B**) PFS in the M2 macrophage-rich group and the M2 macrophage-poor group according to Kaplan–Meier analysis (cutoff = 8.94/HPF). Data were analyzed using a log-rank test. (**C**) Cervical cancer cell lines were co-cultured or not (control) with M2 macrophages for 24 h, followed by 8 Gy radiation. Cell cycle analysis was performed 24 h after radiation, and DNA contents were measured by propidium iodide staining followed by flow cytometry. Data are shown as mean ± SD from three independent experiments. * *p* < 0.05, ** *p* < 0.01 (Student’s *t*-test). (**D**) Cervical cancer cells (SiHa) were co-cultured with M2 macrophages or not (control) for 24 h before 8 Gy radiation. Apoptosis was analyzed by flow cytometry 48 h after radiation. Data represent both propidium iodide and Annexin V staining. Cell populations were gated to determine the apoptotic cell population (Annexin V-positive). Data are shown as mean ± SD from three independent experiments. * *p* < 0.05 (Student’s *t*-test). (**E**) Surviving fractions of cervical cancer cells co-cultured with M1 macrophages or M2 macrophages, or not co-cultured (control) after IR with the indicated doses. (**F**) Representative immunofluorescence images of γ-H2AX foci in cervical cancer cells co-cultured with M2 macrophages or not (control) 24 h after 8 Gy radiation (red, γ-H2AX; blue, DAPI). Data are shown as mean ± SD from three independent experiments. * *p* < 0.05, ** *p* < 0.01 (unpaired Student’s *t* test). (**G**) Western blot showing elevated p-Akt and p-Erk1/2 levels in M2 co-culture groups but no obvious changes in non-co-cultured groups. The whole western blots are shown in File S1.

To evaluate the role of M2 macrophages in cervical cancer, we first detected the expression of CD163, which is considered an M2 macrophage marker [17], in 151 biopsy specimens from patients with cervical cancer who received radical radiotherapy. The CD163 expression levels varied among patients (Figure 1A). A receiver-operating characteristic (ROC) curve was drawn to determine a suitable cut-off value for high-CD163 and low-CD163 expression groups (Figure S1). The cut-off was set to > 8.96 macrophages per high-power field (HPF) of view (sensitivity = 58.2%, specificity = 58.3%). Clinico-pathological features (age, tumor size, pathological type, clinical stage, and lymph node metastasis) were compared between the two groups (Table S1). The lymph node metastasis rate in the high-CD163 expression group (36.1%) was significantly higher than that in the low-CD163 expression group (21.5%) (*p* < 0.05). Furthermore, PFS was lower in the high-CD163 expression group than in the low-CD163 expression group (*p* < 0.05) (Figure 1B), suggesting that M2 macrophages may play a role in the radiosensitivity of cervical cancer. 

To further demonstrate the role of M2 macrophages in the radiosensitivity of cervical cancer, we co-cultured human mononuclear cell line THP-1 cell-derived M2 macrophages (Figure S2A) with cervical cancer cell lines. After radiation, the M2 macrophage co-culture group showed lower levels of G2/M cell cycle arrest (Figure 1C) and apoptosis (Figure 1D), enhanced colony formation ability (Figure 1E), and a lower foci formation frequency (Figure 1F) than cancer cells cultured alone, indicating that the M2 macrophages induced radioresistance in the cervical cancer cells. Further, the phosphorylation levels of Akt and Erk1/2 in the M2 macrophage co-culture group were significantly increased after IR compared to those in the cancer cell cultured alone (Figure 1G), indicating that M2 macrophages may induce cervical cancer cell radioresistance via these pathways.

### 3.2. Macrophages Tend to Be More M2-like Phenotype after High-Dose IR

**Figure 2:**
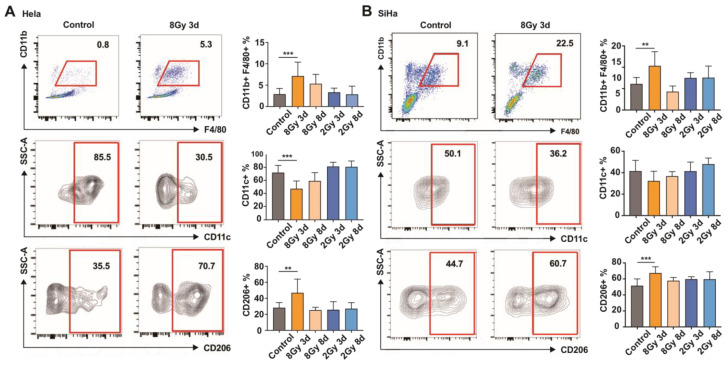
Macrophages tend to be M2 polarized after high-dose IR. Flow-cytometric profile of macrophages in tumor tissues of subcutaneously implanted Hela (**A**) and SiHa (**B**) mouse models of cervical cancer. Tumor tissues were isolated from each group (n = 8) on the 3rd or 8th day after 2 Gy or 8 Gy IR. Macrophages were gated as CD45+/GR-1–/CD11b+/F4/80+ immune cells. CD11c+ M1 and CD206+ M2 were gated on macrophages. Stained cells were subjected to flow cytometry. The data were analyzed using one-way ANOVA. ** *p* < 0.01, *** *p* < 0.001.

To investigate phenotypic changes in macrophages in the TME after different doses of IR, we established subcutaneous xenograft mouse models using HeLa and SiHa cervical cancer cells. When tumor volumes reached 100 mm3, 2 Gy or 8 Gy IR was administered. Tumor tissues were collected for flow cytometry analysis on the 3rd or 8th day after ra-diation. In HeLa model mice, the proportion of CD11b+F4/80+ macrophages was signifi-cantly increased after 8 Gy IR in vivo. Macrophages displayed elevated expression of the M2 marker CD206 on the 3rd day after 8 Gy IR, but not on the 8th day after 8 Gy IR or after 2 Gy radiation (Figure 2A). Similar results were obtained in the SiHa xenograft mouse model (Figure 2B).

### 3.3. CAFs Tend to Promote M2 Polarization of TAMs after High-Dose IR

**Figure 3:**
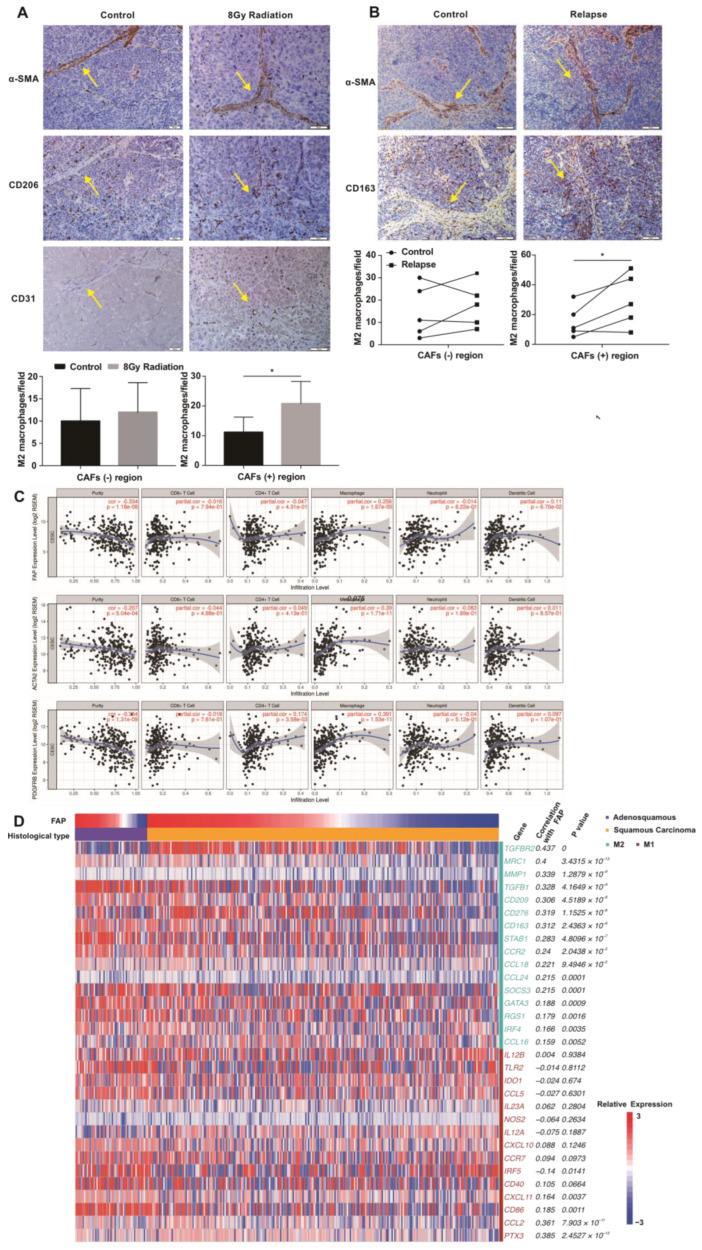
CAFs are associated with M2-like polarization after high-dose IR. Representative images of CD163+ M2 and α-SMA+ CAFs in tumor-bearing mice (**A**) and cervical cancer patients who received brachytherapy (**B**). CAF-positive and -negative regions were assessed in low-power fields. Then, three high-power fields were randomly selected in the same region and CD163+ M2 macrophages were counted. Differences between groups were analyzed using an unpaired Student’s *t*-test. * *p* < 0.05. (**C**) Relationships between CAF-associated genes (FAP, α-SMA (ATGA2), PDGFRB) and the immune infiltration ratio as analyzed using TIMER data (https://cistrome.shinyapps.io/timer/ (accessed on 28 February 2019). (**D**) Heatmap showing the expression of M1/M2-related genes according to histology and ordered by FAP. RSEM-normalized RNA-sequencing log2-transformed data were extracted from TCGA cervical cancer database. The data were analyzed using Spearman’s rank correlation test.

To explore the mechanism of phenotypic transformation of TAMs after radiotherapy, we irradiated macrophages alone (Figure S3A) or after co-culture with irradiated HeLa (Figure S3B) and SiHa (Figure S3C) cervical cancer cells. However, no obvious change in M2 marker expression was observed. We suspected that interstitial components in cervical cancer tissue may be involved in this process. Immunohistochemical staining of serial mouse tumor sections revealed that M2 macrophages accumulated in CAF-positive areas after high-dose radiotherapy (Figure 3A). Similar findings were made in clinical specimens; the numbers of M2 macrophages in CAF-positive areas in tumors of recurrent cervical cancer patients after radical radiotherapy were significantly higher than those before treatment (Figure 3B). CAFs, one of the most important components of the inter-stitial tissue in cervical cancer, express α-SMA and are able to promote M2 polarization. We found that CAF markers, such as fibroblast activation protein (FAP), ACTA2 (α-SMA), and beta-type platelet-derived growth factor receptor (PDGFRB) were associated with the infiltration of macrophages in cervical cancer in the Tumor Immune Estimation Resource (TIMER) database (Figure 3C). RNA-sequencing data were extracted from The Cancer Genome Atlas (TCGA) cervical cancer database (307 patients), normalized using RSEM and log2-transformed. Correlations between macrophage phenotypic molecules and FAP levels were determined using Spearman’s rank correlation test. FAP levels were positively correlated with M2 macrophage markers, including mannose receptor c-type 1 (MRC1), CD163, CCR2, and CD209 (Figure 3D). On the basis of these results, we hypothesized that CAFs may be more potent in driving the phenotypic transformation of macrophages in the context of high-dose radiotherapy.

**Figure 4:**
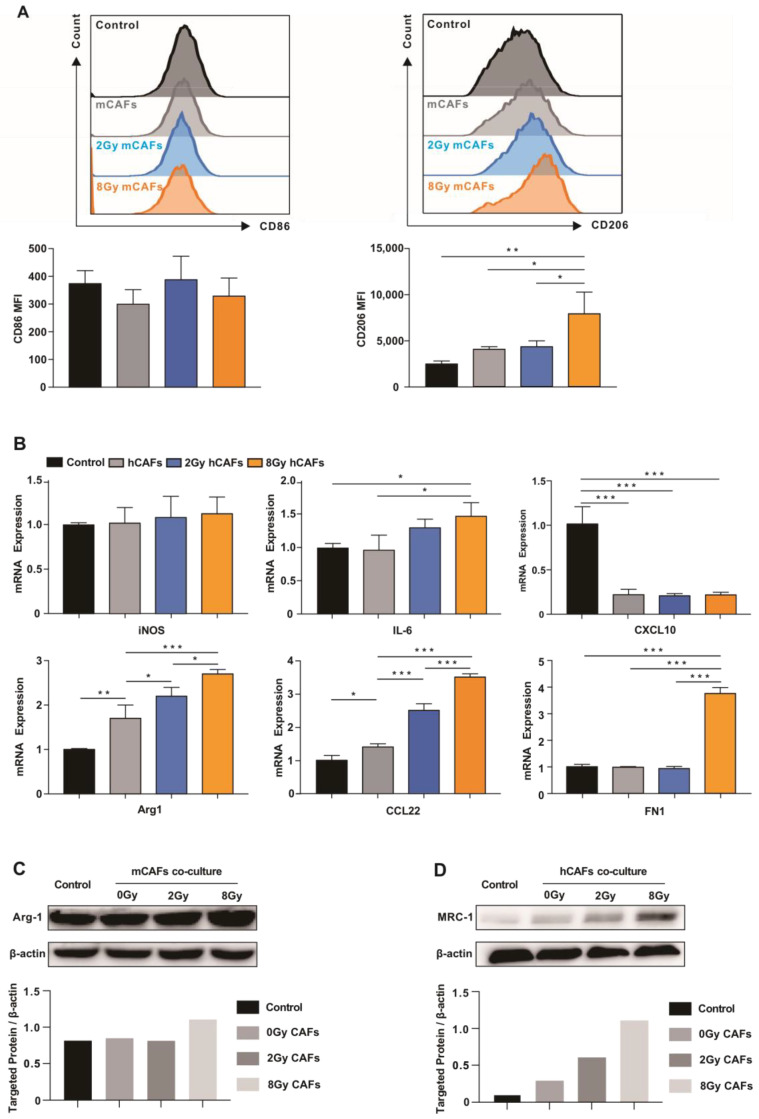
High-dose irradiated CAFs promote macrophage M2 transformation in vitro. Mouse CAFs (mCAFs) or human CAFs (hCAFs) were exposed to 2 or 8 Gy IR to generate irradiated CAFs. The irradiated and non-irradiated mCAFs/hCAFs were immediately co-cultured with BMDMs/THP-1 derived macrophages for 3 days. M0 BMDMs were used as a control. (**A**) Expression of CD206 and CD86 in BMDMs was analyzed by flow cytometry. Data are shown as mean ± SD from three independent experiments. * *p* < 0.05, ** *p* < 0.01, *** *p* < 0.001. (**B**) The expression of various genes related to the macrophage phenotype was determined by RT-qPCR analysis. Data are shown as mean ± SD from three independent experiments. * *p* < 0.05, ** *p* < 0.01, *** *p* < 0.001. (**C**) Western blot analysis of Arg-1 expression. (**D**) Western blot analysis of MRC1 (CD206) expression in PBMC-derived macrophages. The whole western blots are shown in File S1.

CAFs cultured in vitro were subjected to different doses of IR and then co-cultured with M0 macrophages. CD206 expression was higher in M0 BMDMs co-cultured with 8 Gy irradiated CAFs than in M0 BMDMs co-cultured with non-irradiated CAFs, whereas there was no significant change in CD86 expression after IR (Figure 4A). The expression levels of M2 markers Arg-1, CCL22, and FN1 in THP-1 cell-derived macrophages after co-culture with 8 Gy CAFs were higher than those in the control cells (Figure 4B). In addition, Arg-1 expression in M0 BMDMs was increased after co-culture with 8 Gy irradiated CAFs (Figure 4C). MRC-1 expression in M0 peripheral blood mononuclear cell (PBMC)-derived macrophages was increased after co-culture with 8 Gy irradiated CAFs (Figure 4D). Furthermore, high-dose irradiated CAFs suppressed the phagocytic ability of macrophages (Figure S4). Therefore, we hypothesized that CAFs play an important role in high-dose IR-induced macrophage reprogramming in cervical cancer.

### 3.4. CCL2 Plays a Role in Irradiated CAF-Mediated M2-like Polarization 

**Figure 5:**
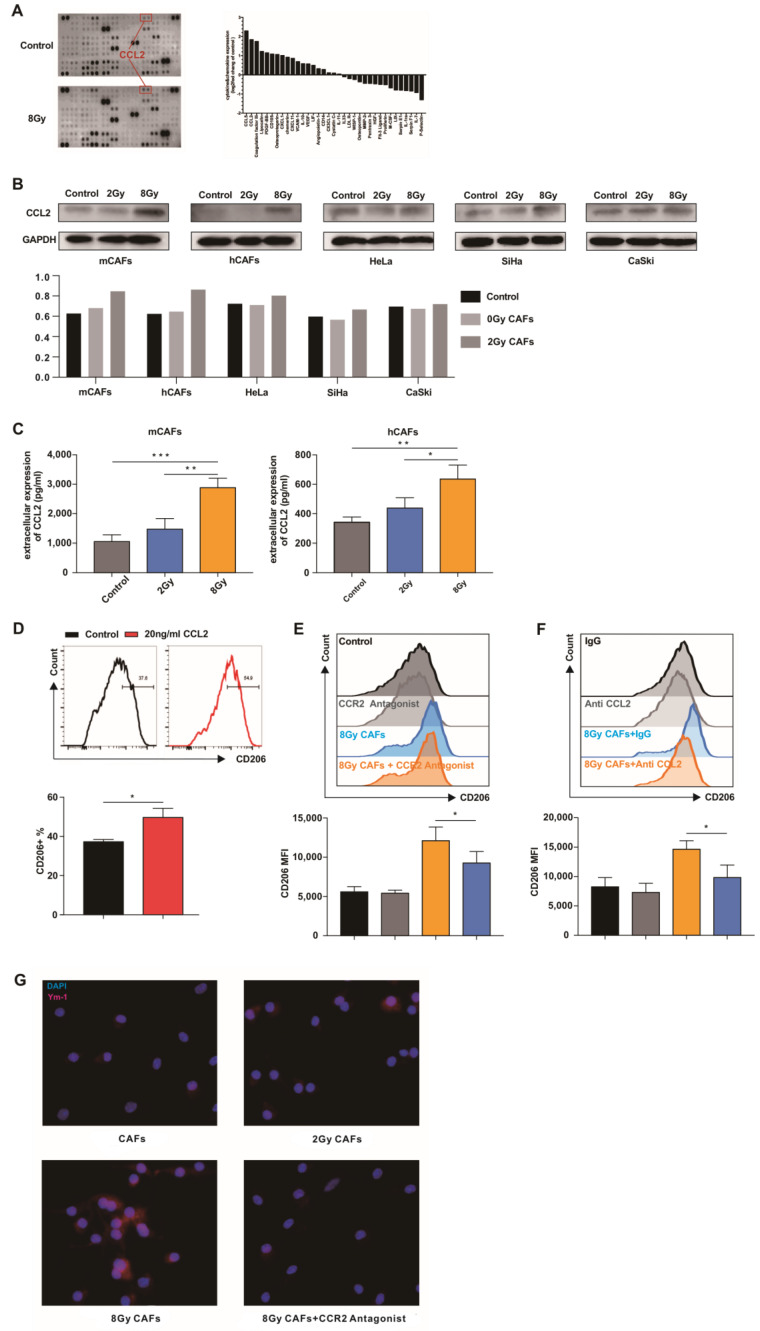
CCL2 is critical for high-dose irradiated CAFs to promote M2 polarization. (**A**) mCAFs were exposed to 8 Gy radiation or not (control), and the supernatant of mCAFs was subjected to multiplex cytokine array analysis 24 h later. Left: a representative blot. Right: quantification of dot intensity of significantly changed cytokines. (**B**) Cervical cancer cell lines and CAFs were irradiated with the indicated doses. After 24 h, CCL2 expression was analyzed by Western blot analysis. (**C**) mCAFs and hCAFs were exposed to the indicated doses of radiation. After 24 h, the supernatant was subjected to ELISA. Data are shown as mean ± SD from three independent experiments. The data were analyzed using one-way ANOVA. * *p* < 0.05, ** *p* < 0.01, *** *p* < 0.001. (**D**) BMDMs were treated with 20 ng/mL CCL2 for 24 h and then subjected to flow cytometry analysis. Data are shown as mean ± SD from three independent experiments. Differences between groups were analyzed using unpaired Student’s *t*-test. * *p* < 0.05. (**E**) M0 BMDMs treated with 10 nM CCR2 antagonist (INCB3344) were co-cultured with irradiated or non-irradiated mCAFs for 3 days. Then, the cells were subjected to flow cytometry analysis to evaluate CD206 expression. Data are shown as mean ± SD from three independent experiments. * *p* < 0.05. (**F**) Anti-CCL2 neutralizing antibody (20 μg/mL) was added to a co-culture system consisting of 8 Gy irradiated mCAFs and M0 BMDMs. The BMDMs were subjected to flow cytometry analysis after 3 days of co-cultivation. Data are shown as mean ± SD from three independent experiments. The data were analyzed using one-way ANOVA. * *p* < 0.05. (**G**) M0 BMDMs were co-cultured with mCAFs exposed to the indicated doses of radiation for 3 days and 10 nM CCR2 antagonist (INCB3344) was added into the 8 Gy irradiated mCAF co-culture system. Immunofluorescence analysis of the expression of Ym-1 (red) in BMDMs. The whole western blots are shown in File S1.

To validate the above hypothesis and unravel the underlying mechanisms, cytokine and chemokine assays were performed to analyze changes in secretory molecules from CAFs after high-dose IR. We found that CCL5, coagulation factor III, and CCL2 were significantly increased in the supernatant of CAFs after high-dose IR (Figure 5A). Treatment of macrophages with exogenous CCL5 or coagulation factor III had no effect on the macrophage phenotype. We then focused on CCL2. Western blot (Figure 5B) and ELISA (Figure 5C) results revealed elevated secretion of CCL2 from CAFs rather than cervical cancer cells after IR with 8 Gy (Figure 5B). After treatment with 20 ng/mL CCL2, CD206 expression in macrophages was increased (Figure 5D). These results indicated that CCL2 promotes macrophage M2 transformation. CCR2 expression was not altered in macrophages co-cultured with irradiated CAFs (Figure S5A), indicating that the M2 phenotypic transformation was mainly due to increased secretion of CCL2 from the CAFs.

To confirm whether CCL2 is required for M2 transformation, we used a CCL2-neutralizing antibody and a CCR2 antagonist to abolish the effect of CCL2 in the co-culture system. Flow cytometry analysis revealed that treatment with these agents partially reversed the effect of irradiated CAFs on macrophages (Figure 5E,F). Ym-1 (chitinase 3) is a known M2 macrophages marker. Immunofluorescence staining showed that Ym-1 expression in co-cultured macrophages was decreased after CCR2 inhibition (Figure 5G). PPARγ is a nuclear receptor that has potent anti-inflammatory properties. Activation of PPARγ primes macrophage M2 polarization. After exogenous administration of CCL2, BMDMs and THP-1-derived macrophages had increased PPARγ expression (Figure S5B,C). Inhibition of PPARγ reversed the effect of CCL2 on macrophage M2 polarization (Figure S5D). Therefore, we concluded that CCL2 mediates high-dose irradiated CAF-mediated M2 transformation via PPARγ.

**Figure 6:**
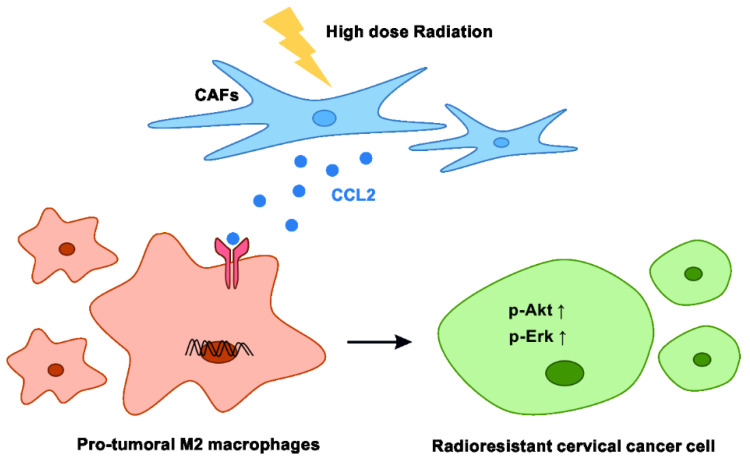
Model summarizing the proposed signaling events among CAFs, TAMs, and cervical cancer cells under high-dose radiation. CAFs secrete an elevated level of CCL2 upon treatment with high-dose radiation. CCL2 promotes pro-tumor transition in macrophages. M2 macrophages induce radioresistance in cervical cancer cells.

In brief, the results indicate that high-dose irradiated CAFs regulate M2 phenotype polarization via increased secretion of CCL2, which subsequently induces radioresistance in cervical cancer (Figure 6).

## 4. Discussion

Radiotherapy has known immunomodulatory effects and is a critical treatment for cervical cancer, with brachytherapy being an essential element for curative-intent treatment. In the present study, we investigated the effect of M2 macrophages on the radiosensitivity of cervical cancer cells and unveiled the time window and the cause of the phenotypic transformation of TAMs in cervical cancer in the context of radiation. 

TAMs have distinct functions depending on the environmental stimuli that affect their phenotype. In past decades, researchers found that M2 macrophage contents correlate with the prognosis of various cancers. Consistent with findings in other studies on breast cancer and head and neck cancer [18,19], we found that M2 macrophages reduced the radiosensitivity of cervical cancer cells. Therefore, the effect of radiation on the macrophage phenotype is crucial for the development of radioresistance in cervical cancer. However, the effects of radiotherapy on macrophage polarization have been inconsistent among studies. After a single high dose of ablation radiotherapy, the innate immune system is activated by inflammatory cytokines such as IL-1, TNF and M-CSF and profibrotic factors such as TGFβ, which may impair T-cell functions and promote tumor progression and recurrence by recruiting M2 macrophages [7,11]. After low-dose radiation, iNos^+^CD68^+^ cells (M1 macrophages) in tumor tissue increase, contributing to vascular normalization, T-cell recruitment, and the inhibition of tumor progression [20]. We reported that TAMs tended to be M2 polarized in the xenograft mouse models of cervical cancer after high-dose irradiation, but not after 2 Gy radiation. Moreover, the phenotypic transformation was dynamic, and its time window was focused on the third day after radiation. 

We also demonstrated that CAFs play a crucial role in the process of phenotypic transformation of TAMs after radiation. It has long been recognized that wound healing is closely related to the mechanism of tumorigenesis, and malignant tumors are described as “non-healing wounds” [21]. In the TME, CAFs play a key role in wound healing. TAMs and CAFs interact with each other and ultimately promote the progression of neuroblastoma and prostate cancer [14,16,22]. However, the immune-modulatory effects of CAFs on macrophages after radiation have rarely been discussed. Berzaghi et al. [23] demonstrated that CAFs isolated from non-small-cell lung cancer tumors promoted changes in M0 macrophages that were in line with both M1 and M2 phenotypes and inhibited pro-inflammatory features of M1 macrophages by suppressing the production of nitric oxide and pro-inflammatory cytokines, migration, and M1 surface marker expression (both in co-culture and in the presence of CAF-conditioned medium). Moreover, CAFs maintained their immunoregulatory effect on macrophages after 1 × 18 Gy or 3 × 6 Gy IR. In accordance with American Brachytherapy Society guidelines, the total high-dose rate of brachytherapy treatment for cervical cancer is 25–30 Gy in 4–5 separate fractions [24]. Therefore, we applied an 8 Gy radiation dose to be consistent with clinical practice. We found that CAFs had an enhanced capacity for promoting the M2 polarization of TAMs after 8 Gy radiation in cervical cancer. Our results diffed from previous findings to some extent, which is likely due to differences in the samples, cancer types and radiation regimens studied. Therefore, further studies are needed to elucidate the relationship between TAMs and CAFs in the context of radiation.

It has been shown that disrupting the interactions between CAFs and TAMs may be useful for improving the efficacy of radiation therapy. As for the detailed molecular mechanisms, it has been reported that CAFs can promote macrophage M2 polarization through immunomodulatory factors, such as M-CSF [25], endosialin [26], IL-6, and GM-CSF [27]. We reasoned that CCL2 may be a key regulator of M2 transformation induced by high-dose irradiated CAFs. CCL2 is an effective chemokine in monocytes, T cells, and NK cells. CCL2 is overexpressed in various cancers and is associated with poor prognosis in breast, colorectal, and thyroid cancers [28], and a lack of CCL2 is associated with increased survival in patients with cervical cancer [29]. The recruitment of TAMs in the TME is generally mediated via the CCL2-CCR axis. As for its effect on macrophage polarization, Roca et al. showed that CCL2 could shift human peripheral blood CD11b+ cells toward a CD206+ M2-polarized phenotype [30], and Sierra-Filardi et al. disclosed an important role for the CCL2-CCR2 axis in regulating macrophage polarization [31]. Consistent with these study findings, we demonstrated that high-dose irradiated CAFs promoted M2 polarization through the secretion of CCL2. Though studies have reported that CCL2 is an important factor in inducing tumor progression, clinical trials targeting CCL2 did not yield promising results [32,33]. The limited curative effect may be ascribed to the negative feedback of CCL2 and the compensation of other cytokines or chemokines after CCL2 inhibition. Therefore, the detailed molecular mechanisms involving CAFs, TAMs and cancer cells remain unraveled. 

This study had some limitations. Immunological studies were performed in xenograft models established using BALB/c-Nu mice, which may differ from syngeneic immunocompetent model mice. Cervical cancer is associated with human papillomavirus infection, and a mature immunocompetent mouse cervical cancer model is currently lacking. Therefore, to investigate TAMs, we used xenograft mouse models, which are widely adopted by researchers [34].

## 5. Conclusions

Our study demonstrated that M2 macrophage associates with the poor prognosis and radioresistance of cervical cancer. Notably, our data indicate that high-dose irradiated CAFs showed an enhanced immunosuppressive effect on macrophage reprogramming via CCL2. Focusing on the relationship between TME components under radiation provides us with a new perspective that may aid in the resolution of radioresistance. 

## Data Availability

Data of TCGA cancer database is available on www.portal.gdc.cancer.gov.

## Supplementary Materials

The following supporting information can be downloaded at: https://www.mdpi.com/article/10.3390/cancers15051620/s1, Table S1: M2 macrophages and clinicopathological features of cervical cancer; Figure S1. ROC curve for the determination of cut-off value for M2 macrophages; Figure S2: Induction of macrophages polarization; Figure S3: Radiation alone or irradiated cervical cancer cell lines couldn’t effect on macrophage’s phenotype; Figure S4: High-dose irradiated CAFs attenuated macrophages’ phagocytosis capacity; Figure S5: CCL2 promote M2 transformation through PPAR-γ; File S1: the whole western blots.

## References

[1] Bray F., Ferlay J., Soerjomataram I., Siegel R.L., Torre L.A., Jemal A. (2018). Global cancer statistics 2018: GLOBOCAN estimates of incidence and mortality worldwide for 36 cancers in 185 countries. CA Cancer J. Clin..

[2] Cohen P.A., Jhingran A., Oaknin A., Denny L. (2019). Cervical cancer. Lancet.

[3] Chargari C., Deutsch E., Blanchard P., Gouy S., Martelli H., Guérin F., Dumas I., Bossi A., Morice P., Viswanathan A.N. (2019). Brachytherapy: An overview for clinicians. CA Cancer J. Clin..

[4] Chen C.-C., Lin J.-C., Jan J.-S., Ho S.-C., Wang L. (2011). Definitive intensity-modulated radiation therapy with concurrent chemotherapy for patients with locally advanced cervical cancer. Gynecol. Oncol..

[5] Kidd E.A., Siegel B.A., Dehdashti F., Rader J.S., Mutic S., Mutch D.G., Powell M.A., Grigsby P.W. (2010). Clinical outcomes of definitive intensity-modulated radiation therapy with fluorodeoxyglucose-positron emission tomography simulation in patients with locally advanced cervical cancer. Int. J. Radiat. Oncol. Biol. Phys..

[6] Xue R., Cai X., Xu H., Wu S., Huang H. (2018). The efficacy of concurrent weekly carboplatin with radiotherapy in the treatment of cervical cancer: A meta-analysis. Gynecol. Oncol..

[7] Barker H.E., Paget J.T.E., Khan A.A., Harrington K.J. (2015). The tumour microenvironment after radiotherapy: Mechanisms of resistance and recurrence. Nat. Rev. Cancer.

[8] Yin Z., Li C., Wang J., Xue L. (2019). Myeloid-derived suppressor cells: Roles in the tumor microenvironment and tumor radiotherapy. Int. J. Cancer.

[9] Falleni M., Savi F., Tosi D., Agape E., Cerri A., Moneghini L., Bulfamante G.P. (2017). M1 and M2 macrophages’ clinicopathological significance in cutaneous melanoma. Melanoma Res..

[10] Jiao X., Zhang S., Jiao J., Zhang T., Qu W., Muloye G.M., Kong B., Zhang Q., Cui B. (2019). Promoter methylation of SEPT9 as a potential biomarker for early detection of cervical cancer and its overexpression predicts radioresistance. Clin. Epigenetics.

[11] Seifert L., Werba G., Tiwari S., Ly N.N.G., Nguy S., Alothman S., Alqunaibit D., Avanzi A., Daley D., Barilla R. (2016). Radiation Therapy Induces Macrophages to Suppress T-Cell Responses Against Pancreatic Tumors in Mice. Gastroenterology.

[12] Groves A.M., Johnston C.J., Misra R.S., Williams J.P., Finkelstein J.N. (2016). Effects of IL-4 on pulmonary fibrosis and the accumulation and phenotype of macrophage subpopulations following thoracic irradiation. Int. J. Radiat. Biol..

[13] Tsai C.-S., Chen F.-H., Wang C.-C., Huang H.-L., Jung S.-M., Wu C.-J., Lee C.-C., McBride W.H., Chiang C.-S., Hong J.-H. (2007). Macrophages from irradiated tumors express higher levels of iNOS, arginase-I and COX-2, and promote tumor growth. Int. J. Radiat. Oncol. Biol. Phys..

[14] Hashimoto O., Yoshida M., Koma Y., Yanai T., Hasegawa D., Kosaka Y., Nishimura N., Yokozaki H. (2016). Collaboration of cancer-associated fibroblasts and tumour-associated macrophages for neuroblastoma development. J. Pathol..

[15] Qiu X., Chen D., Liu Y., Duan S., Zhang F., Zhang Y., Li F., Chen C., Chen Y. (2019). Relationship between stromal cells and tumor spread through air spaces in lung adenocarcinoma. Thorac. Cancer.

[16] Comito G., Giannoni E., Segura C.P., Barcellos-De-Souza P., Raspollini M.R., Baroni G., Lanciotti M., Serni S., Chiarugi P. (2014). Cancer-associated fibroblasts and M2-polarized macrophages synergize during prostate carcinoma progression. Oncogene.

[17] Murray P.J., Wynn T.A. (2011). Protective and pathogenic functions of macrophage subsets. Nat. Rev. Immunol..

[18] Rahal O.M., Wolfe A.R., Mandal P.K., Larson R., Tin S., Jimenez C., Zhang D., Horton J., Reuben J.M., McMurray J.S. (2018). Blocking Interleukin (IL)4- and IL13-Mediated Phosphorylation of STAT6 (Tyr641) Decreases M2 Polarization of Macrophages and Protects Against Macrophage-Mediated Radioresistance of Inflammatory Breast Cancer. Int. J. Radiat. Oncol. Biol. Phys..

[19] Fu E., Liu T., Yu S., Chen X., Song L., Lou H., Ma F., Zhang S., Hussain S., Guo J. (2020). M2 macrophages reduce the radiosensitivity of head and neck cancer by releasing HBEGF. Oncol. Rep..

[20] Klug F., Prakash H., Huber P.E., Seibel T., Bender N., Halama N., Pfirschke C., Voss R.H., Timke C., Umansky L. (2013). Low-dose irradiation programs macrophage differentiation to an iNOS(+)/M1 phenotype that orchestrates effective T cell immunotherapy. Cancer Cell.

[21] Mantovani A., Allavena P., Sica A., Balkwill F. (2008). Cancer-related inflammation. Nature.

[22] Herrera M., Herrera A., Domínguez G., Silva J., García V., García J.M., Gómez I., Soldevilla B., Muñoz C., Provencio M. (2013). Cancer-associated fibroblast and M2 macrophage markers together predict outcome in colorectal cancer patients. Cancer Sci..

[23] Berzaghi R., Ahktar M.A., Islam A., Pedersen B.D., Hellevik T., Martinez-Zubiaurre I. (2019). Fibroblast-Mediated Immunoregulation of Macrophage Function Is Maintained after Irradiation. Cancers.

[24] Romano K.D., Hill C., Trifiletti D.M., Peach M.S., Horton B.J., Shah N., Campbell D., Libby B., Showalter T.N. (2018). High dose-rate tandem and ovoid brachytherapy in cervical cancer: Dosimetric predictors of adverse events. Radiat. Oncol..

[25] Zhang A., Qian Y., Ye Z., Chen H., Xie H., Zhou L., Shen Y., Zheng S. (2017). Cancer-associated fibroblasts promote M2 polarization of macrophages in pancreatic ductal adenocarcinoma. Cancer Med..

[26] Yang F., Wei Y., Han D., Li Y., Shi S., Jiao D., Wu J., Zhang Q., Shi C., Yang L. (2020). Interaction with CD68 and Regulation of GAS6 Expression by Endosialin in Fibroblasts Drives Recruitment and Polarization of Macrophages in Hepatocellular Carcinoma. Cancer Res..

[27] Cho H., Seo Y., Loke K.M., Kim S.-W., Oh S.-M., Kim J.-H., Soh J., Kim H.S., Lee H., Kim J. (2018). Cancer-Stimulated CAFs Enhance Monocyte Differentiation and Protumoral TAM Activation via IL6 and GM-CSF Secretion. Clin. Cancer Res..

[28] Mantovani A., Sica A. (2010). Macrophages, innate immunity and cancer: Balance, tolerance, and diversity. Curr. Opin. Immunol..

[29] Zijlmans H.J., Fleuren G.J., Baelde H.J., Eilers P.H., Kenter G.G., Gorter A. (2006). The absence of CCL2 expression in cervical carcinoma is associated with increased survival and loss of heterozygosity at 17q11.2. J. Pathol..

[30] Roca H., Varsos Z.S., Sud S., Craig M.J., Ying C., Pienta K.J. (2009). CCL2 and interleukin-6 promote survival of human CD11b+ peripheral blood mononuclear cells and induce M2-type macrophage polarization. J. Biol. Chem..

[31] Sierra-Filardi E., Nieto C., Domínguez-Soto Á., Barroso R., Sánchez-Mateos P., Puig-Kroger A., López-Bravo M., Joven J., Ardavín C., Rodríguez-Fernáandez J.L. (2014). CCL2 shapes macrophage polarization by GM-CSF and M-CSF: Identification of CCL2/CCR2-dependent gene expression profile. J. Immunol..

[32] Sandhu S.K., Papadopoulos K., Fong P.C., Patnaik A., Messiou C., Olmos D., Wang G., Tromp B.J., Puchalski T.A., Balkwill F. (2013). A first-in-human, first-in-class, phase I study of carlumab (CNTO 888), a human monoclonal antibody against CC-chemokine ligand 2 in patients with solid tumors. Cancer Chemother. Pharmacol..

[33] Brana I., Calles A., Lorusso P.M., Yee L.K., Puchalski T.A., Seetharam S., Zhong B., De Boer C.J., Tabernero J., Calvo E. (2015). Carlumab, an anti-C-C chemokine ligand 2 monoclonal antibody, in combination with four chemotherapy regimens for the treatment of patients with solid tumors: An open-label, multicenter phase 1b study. Target. Oncol..

[34] Liu N., Ma M., Qu N., Wang R., Chen H., Hu F., Gao S., Shan F. (2020). Low-dose naltrexone inhibits the epithelial-mesenchymal transition of cervical cancer cells in vitro and effects indirectly on tumor-associated macrophages in vivo. Int. Immunopharmacol..

