# Supplementary material for: Cancer-Associated Fibroblasts Exposed to High-Dose Ionizing Radiation Promote M2 Polarization of Macrophages, Which Induce Radiosensitivity in Cervical Cancer
Source: Cancers (Basel). 2023 Mar 6;15(5):1620. doi: 10.3390/cancers15051620 (PMC10001412; doi:10.3390/cancers15051620)
Supplement: Supplementary file 1 [file cancers-15-01620-s001.zip › Figures S1-S5.pdf]

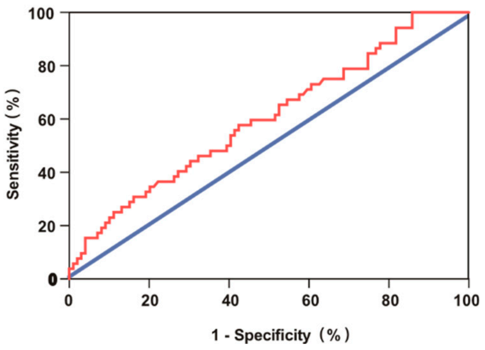

p

Supplementary Figure S1.ROC curve for the determination of cut-off value for M2 macrophages.

The number of $CD163^+$ M2 macrophages was set as a predictor, and disease progression was used as the outcome. The area under the curve is 0.602 (p=0.04). The number of macrophages at the maximum Youden Index (Youden Index = Sensitivity + Specificity -1) is 8.96 per high-power field of view (sensitivity = 58.2%, specificity = 58.3%).

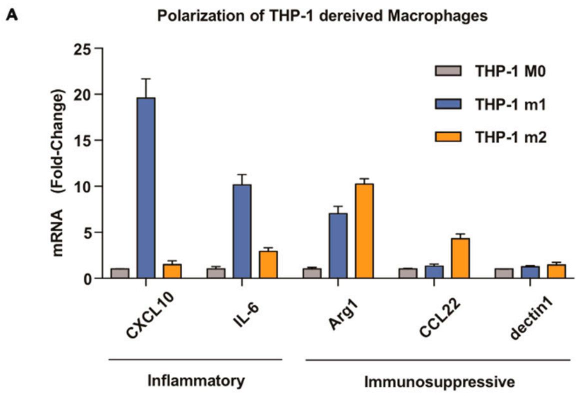

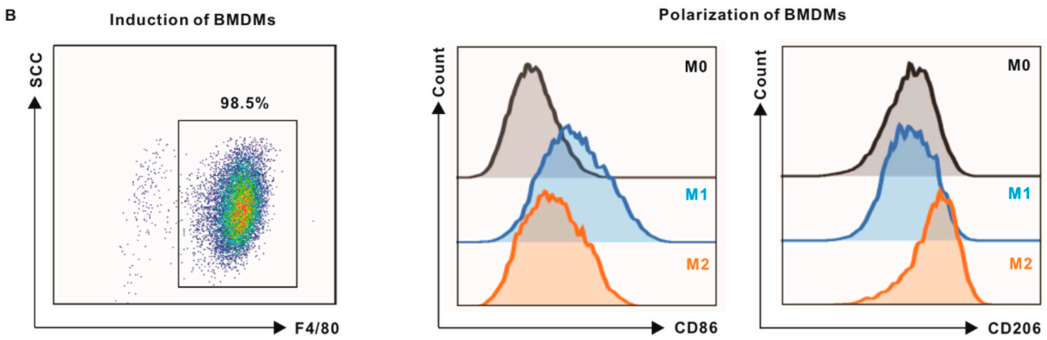

Supplementary Figure S2.Induction of macrophages polarization.

A. THP-1-derived Macrophages were subjected to RT-PCR after transformed into M1 or M2 phenotype. Transcription levels of markers that indicate M2 polarization (Arg-1, CCL22) were elevated in IL-4+IL-13 treated THP-1 M2 macrophages than in unstimulated macrophages (THP-1 M0) and THP-1 M1 macrophages ($p < 0.05$). Markers that indicate M1 polarization (CXCL10, IL-6) was greater in IFN-γ+LPS treated THP-1 M1 macrophages than that in unstimulated macrophages and THP-1 M2 macrophages. Statistic differences were analyzed using one-way ANOVA test. B. Representative figure of the expression of F4/80 in M0 BMDMs (left). Representative figures of flow cytometry analysis after the polarization of M0 BMDMs (right). M1 BMDMs showed enhanced expression of CD86 (M1 marker) than M2 BMDMs and M0 BMDMs, M2 BMDMs showed enhanced expression of CD206 (M2 marker) than M1 BMDMs and M0 BMDMs.

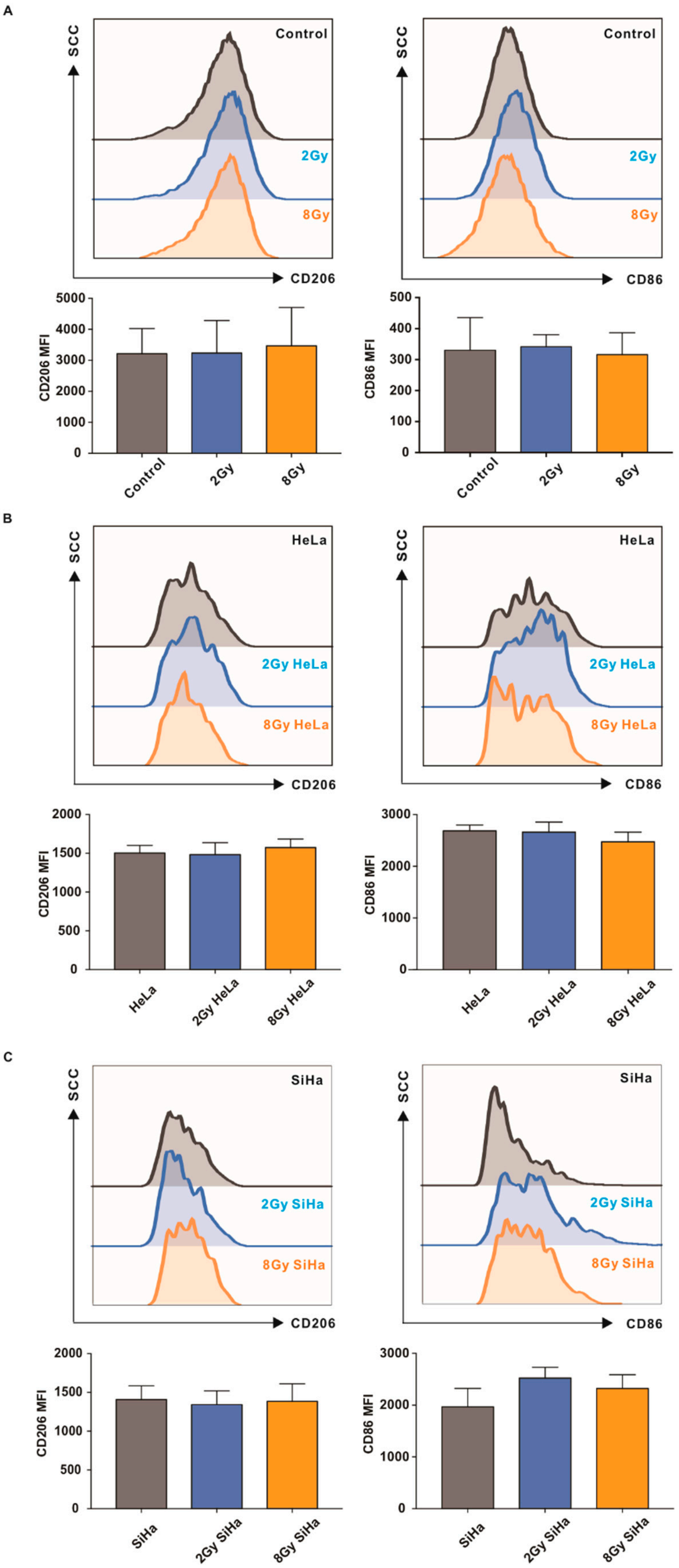
A
SCC
Control
2Gy
8Gy
CD206
CD86
CD206 MFI
CD86 MFI
Control
2Gy
8Gy
B
HeLa
2Gy HeLa
8Gy HeLa
C
SiHa
2Gy SiHa
8Gy SiHa

Supplementary Figure S3. Radiation alone or irradiated cervical cancer cell lines couldn't effect on macrophage's phenotype.

A. M0 BMDMs were irradiated with the indicated doses (0 Gy was set as the control group). Expressions of CD86 and CD206 were evaluated by flow cytometry analysis on the 3rd day after radiation. Data are shown as mean ± SD from three independent experiments. B. M0 PBMC was co-cultured with irradiated or non-irradiated (control) cervical cancer cells HeLa or SiHa for 3 days. Macrophages were collected to evaluate the expressions of CD86 and CD206 by flow cytometry analysis. Data are shown as mean ± SD from three independent experiments. Statistic differences were analyzed using one-way ANOVA test.

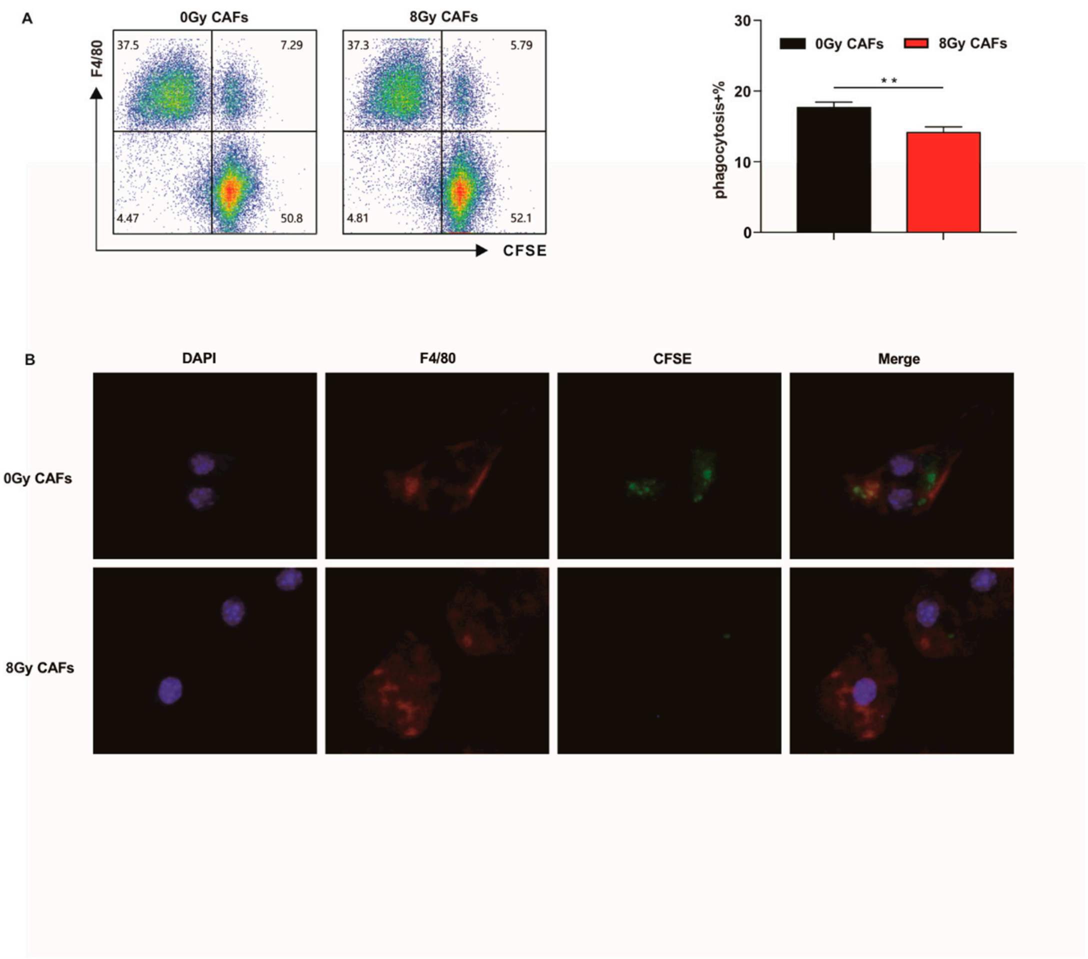

Supplementary Figure S4. High-dose irradiated CAFs attenuated macrophages' phagocytosis capacity.

M0 BMDMs were co-cultured with 8 Gy irradiated mCAFs for 3 days or not (control). Macrophages were labeled with F4/80 and cancer cells were stained with CFSE. The phagocytosis capacity of BMDMs was evaluated by A. flow cytometry analysis and B. immunofluorescence. Data are shown as mean ± SD from three independent experiments. Statistic differences were analyzed using unpaired Student's t test. **$p <$ 0.01

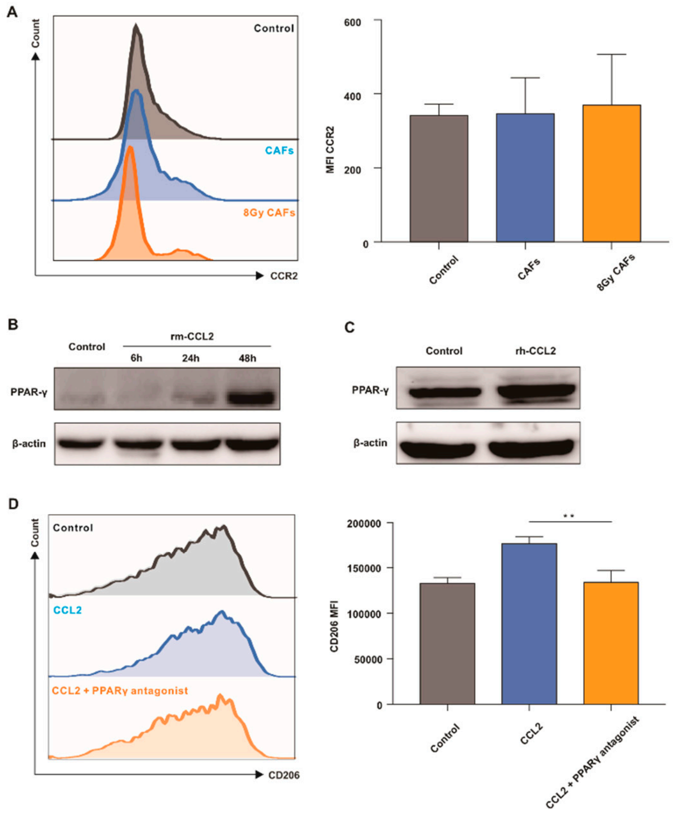

Supplementary Figure S5. CCL2 promote M2 transformation through PPAR-γ.

A. After cultured with irradiated or non-irradiated mCAFs for 3 days, M0 BMDMs were subjected to flow cytometry analysis to evaluate the expression of CCR2. Data are shown as mean ± SD from three independent experiments. B. M0 BMDMs were treated with 20 ng/mL rm-CCL2 and the expression of PPAR-γ was analyzed by western blot. C. THP-1 derived macrophages were treated with 20 ng/mL rh-CCL2 and the expression of PPAR-γ was analyzed by western blot. D. PPAR-γ antagonist (5nM) was added into the medium of M0 BMDMs, followed by treating M0 BMDMs with 20 ng/mL rm-CCL2 for 24h. Expressions of CD206 were evaluated by flow cytometry analysis. Data are shown as mean ± SD from three independent experiments. **$p < 0.01$. C. THP-1 derived macrophages were treated with 20ng/ml rh-CCL2 and the expression of PPAR-γ was analyzed by western blot (up). PPAR-γ antagonist (5nM) was added into the medium of

THP-1 M0, followed by treating THP-1 M0 with 20 ng/mL rh-CCL2 for 24h. Transcription levels of macrophage phenotypic markers such as iNOS, CXCL10, CCL22, FN1, and Dectin were analyzed by RT-PCR. Data are shown as mean ± SD from three independent experiments. Statistic differences were analyzed using one-way ANOVA test. *$p < 0.05$, **$p < 0.01$.
