# Supplementary material for: Cancer-Associated Fibroblasts Exposed to High-Dose Ionizing Radiation Promote M2 Polarization of Macrophages, Which Induce Radiosensitivity in Cervical Cancer
Source: Cancers (Basel). 2023 Mar 6;15(5):1620. doi: 10.3390/cancers15051620 (PMC10001412; doi:10.3390/cancers15051620)
Supplement: Supplementary file 1 [file cancers-15-01620-s001.zip › Table S1.pdf]

Table S1. M2 macrophages and clinicopathological features of cervical cancer

	Low M2 n=79	High M2 n=72	t/$\chi 2$	P
**Age**	57.63±9.83	59.15±9.03	-0.990	0.828
**Tumor size > 4cm**	46 (58.2%)	50 (69.4%)	2.047	0.153
**Lymph node positive**	17 (21.5%)	26 (36.1%)	3.938	0.047
**Distant metastasis**	9 (11.4%)	11 (15.3%)	0.495	0.482
**FIGO (2009)**				
IA-IIA	13 (16.5%)	7 (9.7%)	1.486	0.223
IIB-V	66 (83.5%)	65 (90.3%)		
**Pathological pattern**				
adenocarcinoma	3 (3.8%)	8 (11.1%)	2.983	0.084
squamous carcinoma	76 (96.2%)	64 (88.9%)		
