# Supplementary figures and images for: Cancer-Associated Fibroblasts Exposed to High-Dose Ionizing Radiation Promote M2 Polarization of Macrophages, Which Induce Radiosensitivity in Cervical Cancer
Source: Cancers (Basel). 2023 Mar 6;15(5):1620. doi: 10.3390/cancers15051620 (PMC10001412; doi:10.3390/cancers15051620)

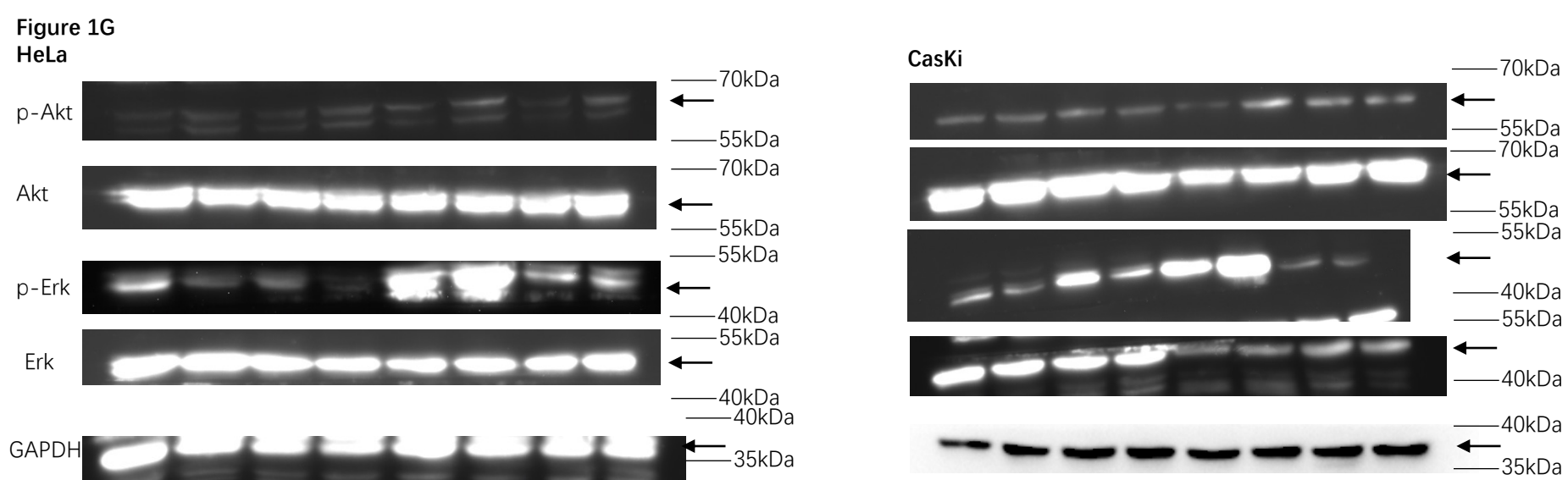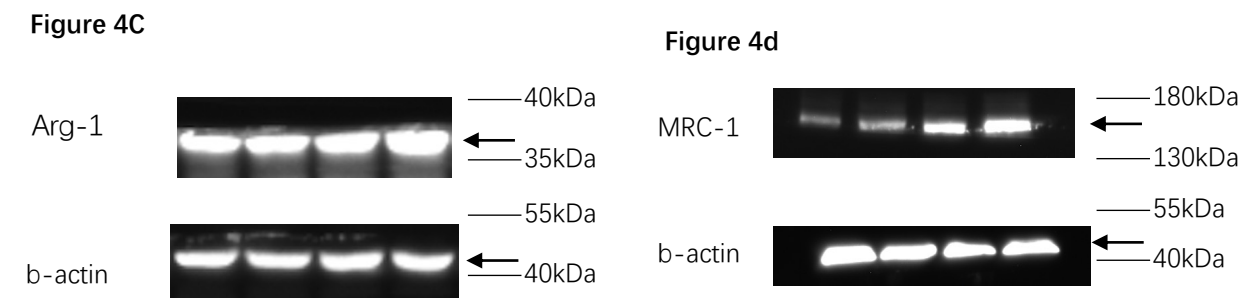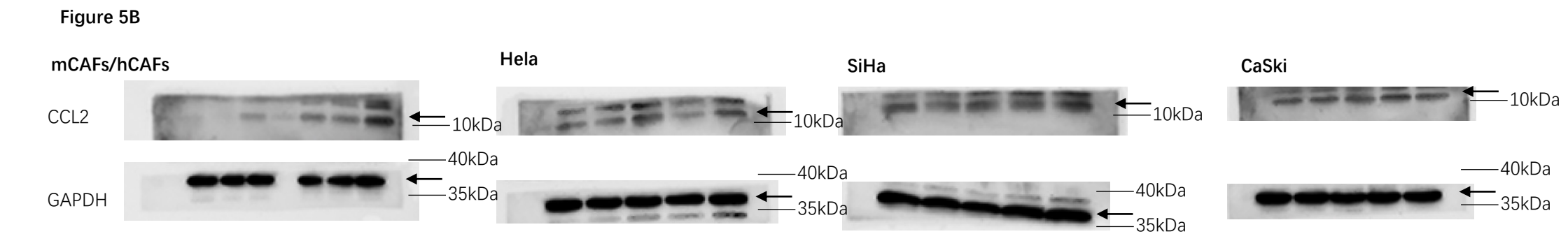

Supplement: Supplementary file 1 [file cancers-15-01620-s001.zip › file S1.pdf]
